# Amyloid Precursor-like Protein 2 Expression Increases during Pancreatic Cancer Development and Shortens the Survival of a Spontaneous Mouse Model of Pancreatic Cancer

**DOI:** 10.3390/cancers13071535

**Published:** 2021-03-26

**Authors:** Brittany J. Poelaert, Shelby M. Knoche, Alaina C. Larson, Poomy Pandey, Parthasarathy Seshacharyulu, Nuzhat Khan, H. Carlo Maurer, Kenneth P. Olive, Yuri Sheinin, Rizwan Ahmad, Amar B. Singh, Surinder K. Batra, Satyanarayana Rachagani, Joyce C. Solheim

**Affiliations:** 1 Eppley Institute for Research in Cancer & Allied Diseases and the Fred & Pamela Buffett Cancer Center, University of Nebraska Medical Center, Omaha, NE 68198, USA; bpoelaert@georgiamune.com (B.J.P.); shelby.knoche@unmc.edu (S.M.K.); alaina.larson@unmc.edu (A.C.L.); poomy.pandey@ki.se (P.P.); nuzkhan75@gmail.com (N.K.); 2 Department of Biochemistry & Molecular Biology and the Fred & Pamela Buffett Cancer Center, University of Nebraska Medical Center, Omaha, NE 68198, USA; p.seshacharyulu@unmc.edu (P.S.); rizwan.ahmad@unmc.edu (R.A.); amar.singh@unmc.edu (A.B.S.); sbatra@unmc.edu (S.K.B.); srachagani@unmc.edu (S.R.); 3 Columbia University Department of Medicine and the Herbert Irving Comprehensive Cancer Center, Columbia University Medical Center, New York, NY 10032, USA; carlo.maurer@tum.de (H.C.M.); kpo2104@cumc.columbia.edu (K.P.O.); 4 Department of Pathology and Microbiology and the Fred & Pamela Buffett Cancer Center, Omaha, NE 68198, USA; ysheinin@mcw.edu

**Keywords:** amyloid precursor-like protein 2, mouse model, pancreatic cancer

## Abstract

**Simple Summary:**

As pancreatic cancer is a disease with a high fatality rate, a better understanding of how it develops and the identification of new potential targets for its treatment are greatly needed. In this current study, we showed that the expression of amyloid precursor-like protein 2 (APLP2) in pancreatic cancer epithelial cells is higher than in precursor lesion epithelial cells, thus indicating that APLP2 increases during human pancreatic cancer development. We also generated a new mouse model that demonstrated the deletion of APLP2 expression specifically within the pancreas prolongs survival and decreases metastasis for mice with pancreatic cancer. Taken together, these findings open a new avenue toward comprehending and treating pancreatic cancer.

**Abstract:**

In the United States, pancreatic cancer is a major cause of cancer-related deaths. Although substantial efforts have been made to understand pancreatic cancer biology and improve therapeutic efficacy, patients still face a bleak chance of survival. A greater understanding of pancreatic cancer development and the identification of novel treatment targets are desperately needed. Our analysis of gene expression data from patient samples showed an increase in amyloid precursor-like protein 2 (APLP2) expression within primary tumor epithelium relative to pancreatic intraepithelial neoplasia (PanIN) epithelial cells. Augmented expression of APLP2 in primary tumors compared to adjacent stroma was also observed. Genetically engineered mouse models of spontaneous pancreatic ductal adenocarcinoma were used to investigate APLP2′s role in cancer development. We found that APLP2 expression intensifies significantly during pancreatic cancer initiation and progression in the *LSL-Kras^G12D/+^*; *LSL-Trp53^R172H/+^*; *Pdx-1-Cre* (KPC) mouse model, as shown by immunohistochemistry analysis. In studies utilizing pancreas-specific heterozygous and homozygous knockout of APLP2 in the KPC mouse model background, we observed significantly prolonged survival and reduced metastatic progression of pancreatic cancer. These results demonstrate the importance of APLP2 in pancreatic cancer initiation and metastasis and indicate that APLP2 should be considered a potential therapeutic target for this disease.

## 1. Introduction

Pancreatic cancer is one of the leading causes of cancer-related deaths in the United States, and patients with this illness face a dismally low 5-year survival rate [1]. More in-depth understanding of pancreatic cancer progression and the development of efficacious, targeted strategies are necessary. Amyloid precursor-like protein 2 (APLP2), a type 1 transmembrane glycoprotein, is a member of the amyloid precursor protein family, and it is expressed throughout the body at varying levels [2,3,4]. Previous work from our laboratory and other research groups has shown that APLP2 is increased in expression in a variety of human cancers, including Ewing sarcoma, breast cancer, and pancreatic cancer [5,6,7,8,9,10]. Results from our laboratory demonstrated that the transfection of pancreatic cancer cells in vitro with APLP2 short interfering RNA (siRNA) reduced their growth, and transfection with either APLP2 short hairpin RNA (shRNA) or siRNA reduced their migration [6,10]. Our previous studies also showed that knockdown of APLP2 by APLP2-specific shRNA significantly inhibited xenograft pancreatic tumor growth and reduced metastasis to the intestine, diaphragm, and kidney in athymic nude mice [10]. These published findings support a role for APLP2 in pancreatic cancer pathogenesis and also raise the question of whether APLP2 contributes to pancreatic cancer development.

Our current findings show an increase in APLP2 expression in human patient pancreatic adenocarcinoma epithelial cells compared to precursor pancreatic intraepithelial neoplasia (PanIN) lesions (as well as stromal cells), which indicates a correlation between the level of APLP2 expression and the development of pancreatic cancer. Accordingly, we also demonstrated that APLP2 is more highly expressed in tumor specimens acquired from the *LSL-Kras^G12D/+^*; *LSL-Trp53*^R172H/+^; *Pdx-1-Cre* (KPC) mouse model [11,12] than in normal mouse pancreas tissue. To study the role of APLP2 in pancreatic cancer initiation and progression, we generated a variant of the KPC model with a conditional, pancreas-specific knockout of APLP2, thus allowing for longitudinal investigation of the impact of APLP2 on pancreatic cancer development. Findings from this novel mouse model show a significant improvement in the survival of KPC mice with pancreas-specific hetero- or homozygous knockout of APLP2. Overall, our findings demonstrate the contribution of APLP2 as a potentiating factor in pancreatic cancer development and progression, and thus support future studies to evaluate APLP2 as a preventative or therapeutic target.

## 2. Results

### 2.1. APLP2 mRNA Expression Is Higher in Human Primary Pancreatic Tumors Relative to PanIN Epithelial and Stromal Cells

**Figure 1:**
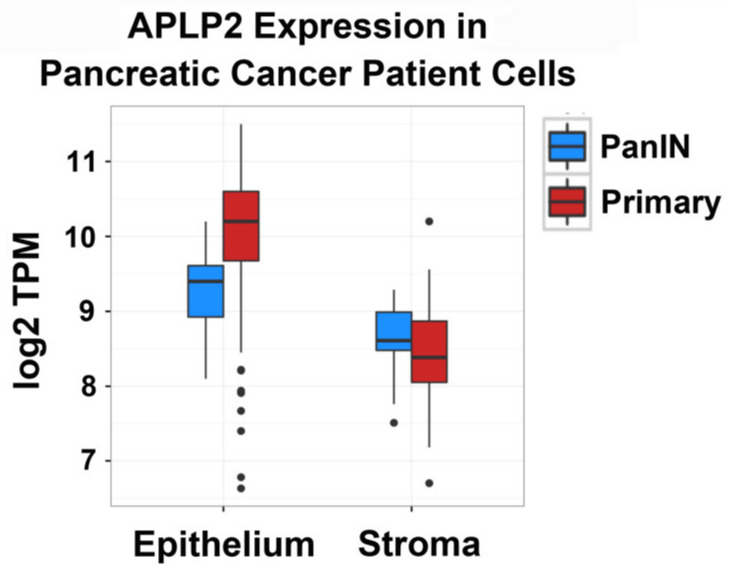
Human primary tumor epithelial cells highly express amyloid precursor-like protein 2 (APLP2). By RNA-Seq analysis, human primary tumor epithelial cells were demonstrated to express significantly more APLP2 than human pancreatic intraepithelial neoplasia (PanIN) epithelial or stromal cells. TPM = Transcripts Per Million.

We investigated the expression of APLP2 in human patient samples of pancreatic adenocarcinoma using RNA-Seq technology [13,14]. Relevant data from the analysis of the expression patterns of marker genes that we used for epithelial cells and stromal cells in our set of human tissue samples have been shown in a previous report published by some members of our research group [13]. As was noted in this publication, the marker genes for epithelium were KRT19, EPCAM, and CDH1. For stroma, the marker genes used were VWF, ENG, and CDH5 (for endothelial cells), DCN, ACTA2, and FAP (for cancer-associated fibroblasts), and PTPRC, CD4, and CD163 (for leukocytes) [13]. As displayed in Figure 1 and Table 1, human primary tumor epithelial cells have increased expression of APLP2 compared to human PanIN epithelial cells, indicating a rise in APLP2 as the lesion progressed from PanIN to a tumor (Table S1 lists the data for all the individual RNA-Seq values from which the mean values shown in Table 1 were derived). In addition, we observed higher APLP2 expression in epithelial cell samples compared to stromal cell samples. These data complement our earlier reports that showed APLP2 protein expression is elevated in human pancreatic primary tumors and metastases in relation to normal pancreas tissue [8,10].

### 2.2. Murine KPC Primary Pancreatic Tumor Tissue Has Increased Expression of APLP2 Protein Compared to PanINs

**Figure 2:**
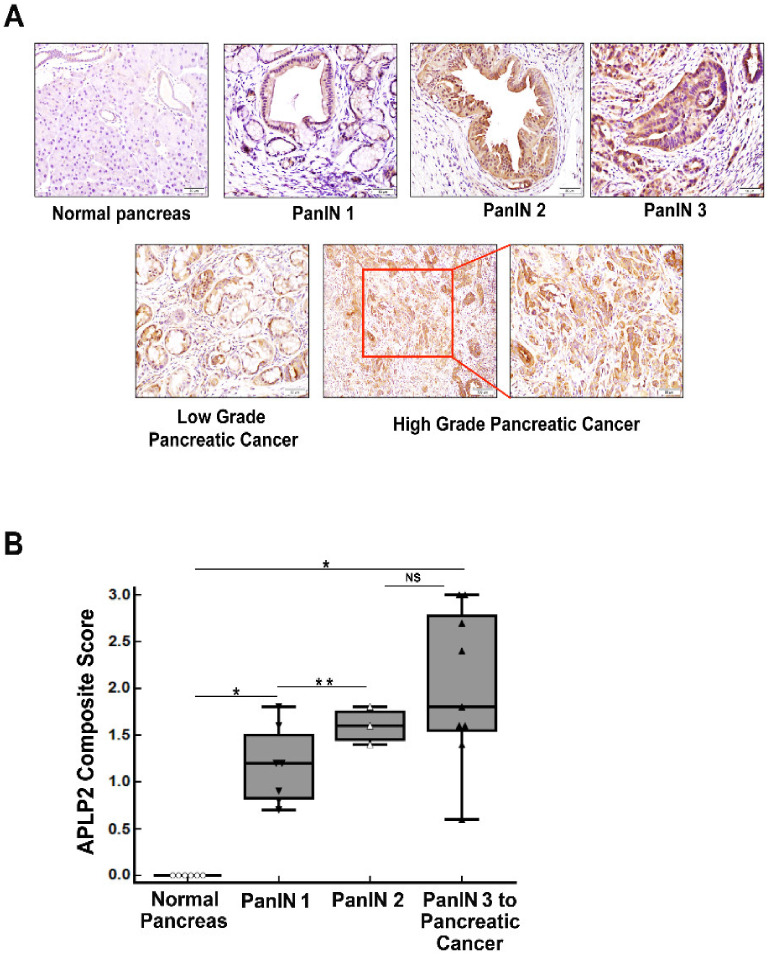
APLP2 increases with progression to pancreatic adenocarcinoma in a mouse model. Immunohistochemistry was used to analyze the expression of APLP2 in tissue samples from 20-week-old KPC mice. (**A**) APLP2 staining was absent in normal pancreas tissues. PanIN 1, 2, and 3 displayed weak to strong staining of APLP2 in the cytoplasm. Both low- and high-grade pancreatic cancer samples with intensely positive cytoplasmic APLP2 staining >80% are shown. A scale bar is displayed in the lower right corner of each section. For all sections except the bottom middle one, the scale bar represents 50 μm. For the bottom middle section, the scale bar represents 100 μm. (**B**) The expression of murine APLP2 in normal and pancreatic tissue is graphically represented in a box plot, which shows the immunohistochemistry data based on H-scores. These scores indicate a statistically significant increase in APLP2 expression in murine PanIN 1 (* *p* < 0.05) compared to the normal murine pancreas and in PanIN 2 compared to PanIN 1 (** *p* < 0.01). APLP2 expression was significantly greater in PanIN 3/pancreatic cancer relative to the normal pancreas (* *p* < 0.05). NS = Not Significant. The staining was scored according to intensity on a 0–3 scale (0 = negative, 1 = weak, 2 = moderate, 3 = strong). The percentage of cells stained for murine APLP2 was evaluated based on the absolute stain (i.e., 10–100% is scored as 0.1 to 1). The Histo-score (H-score) was calculated by multiplying the staining intensity by the percentage of cells positive for APLP2 expression. Statistical analysis was performed using the Student’s *t*-test.

By immunohistochemistry, we also analyzed the expression of murine APLP2 in normal and pancreatic adenocarcinoma tissues acquired from KPC mice (Figure 2). Minimal to no expression of APLP2 was observed in normal pancreas tissue (*n* = 6). However, we saw significant increases in APLP2 staining during disease progression (*n* = 7) relative to the normal pancreas. Further classification of the tissues into developmental stages of the disease (PanIN 1, PanIN 2, PanIN 3, low-grade carcinoma, and high-grade carcinoma) revealed weak to moderate staining in PanIN 1 lesions (with 60–70% of the cells displaying immunoreactivity). PanIN 2 lesions exhibited moderate staining for APLP2, while PanIN 3 and pancreatic adenocarcinoma samples showed strong staining of APLP2 with ~90–100% of cells positively stained (particularly ductal epithelial cells). Overall, we have found that APLP2 protein expression increases as pancreatic cancer develops in KPC mice.

### 2.3. Loss of APLP2 Specifically in the Pancreas Prolongs the Survival of KPC Mice

To gain a deeper understanding of the role of APLP2 in pancreatic cancer development, we generated a new variant of the KPC mouse model that had APLP2 deficiency in the pancreas. To begin, a strain with conditional potential for mouse *Aplp2* gene deletion (the *Aplp2^tm1a/tm1a^* strain) was crossed with the *FLPo-10* mouse strain (which expresses FLP recombinase) to generate *Aplp2^tm1c/+^* animals. The *Aplp2^tm1c/+^* (and *Aplp2^tm1c/tm1c^* mice generated from their interbreeding) are able to grow and reproduce normally, and they have no apparent abnormal phenotypic or developmental characteristics. To produce quadruple mutant animals, *LSL-Aplp2^tmi1c/tm1c^* mice were crossed with *LSL-Kras^G12D/+^*; *LSL-Trp53*^R172H/+^ mice, followed by the crossing of *LSL-Kras^G12D/+^*; *LSL-Trp53*^R172H/+^; *LSL-Aplp2^tmi1c/tm1c^* mice with *Pdx-1-Cre* mice.

**Figure 3:**
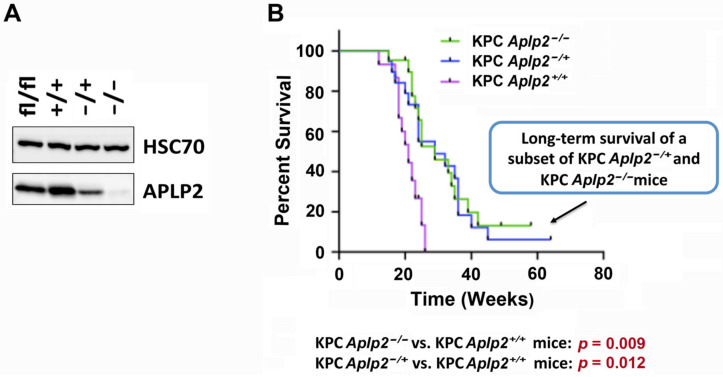
Novel variants of KPC mice with pancreas-specific APLP2 heterozygous or homozygous loss exhibited prolonged survival. (**A**) Expression of APLP2 in pancreatic tissue samples from mouse strains. Immunoblotting to detect HSC70 as a control (~70 kD), as well as the APLP2 C-terminus (~22 kD), was performed on pancreas tissue lysates from KPC mice with floxed (fl/fl), wild-type (+/+), heterozygous deletion (−/+), or homozygous deletion (−/−) of APLP2. The densitometry readings and intensity ratios for these bands are shown in Table S2. (**B**) Pancreas-specific loss of APLP2 prolongs the survival of KPC mice. The graph displays cumulative survival time in weeks with Week 0 indicating birth. The mice were monitored at least 3 times weekly and euthanized per Institutional Animal Care and Use Committee (IACUC) guidelines. Mean survival time +/− standard error of the mean was determined for each group.

APLP2 protein expression in pancreas tissues from quadruple mutant mice was monitored by immunoblotting of tissue lysates obtained from genotyped KPC mice with floxed *Aplp2* (fl/fl), wild-type *Aplp2* (+/+), heterozygous *Aplp2* deletion (−/+), or homozygous *Aplp2* deletion (−/−), using an antibody specific for a sequence in the C-terminal region of APLP2 (Figure 3A and Figure S1). APLP2 normally undergoes secretase cleavage within cells [9], and thus the expression of APLP2 can be confirmed by the detection of an APLP2 cleavage fragment. The expression of APLP2 protein was highest in the floxed and wild-type pancreas tissues (Figure 3A). APLP2 expression was lower in the pancreas tissue from heterozygous pancreas-specific *Aplp2* knockout (−/+), and little to no APLP2 protein was detectable in the pancreas of the homozygous knockout (−/−). Thus, the decrease in APLP2 expression was further potentiated by the loss of the second allele, as observed by the comparison of the immunoblotting results from the heterozygous and homozygous knockouts (Figure 3A).

The KPC mice with homozygous or heterozygous pancreas-specific knockout of APLP2, along with KPC (APLP2 wild-type) mice, were monitored for signs of health or disease (ability to eat and drink, mobility, appearance, palpable tumors or ascites, and duration of survival). Loss or deficiency of APLP2 in the pancreas did not hinder viability, fertility, development, or behavior. When assessing the survival distributions (by Kaplan-Meier survival analysis), we observed a significant improvement in the mean survival time of the KPC mice without pancreas APLP2 expression (Figure 3B). Diminished, but not absent, APLP2 expression in the pancreas (in the heterozygotes) also resulted in a similar significant lengthening of the mean survival time, relative to the KPC mice that have wild-type APLP2 expressed in the pancreas (Figure 3B). The KPC-*Aplp2*^+*/*+^ mouse cohort’s mean survival was 20.8 weeks ± 1.2, compared to a mean survival of 30.7 weeks ± 2.9 for KPC-Panc-*Aplp2^+/−^* mice and 31.0 weeks ± 2.8 for KPC-Panc-*Aplp2^−/−^* mice.

**Figure 4:**
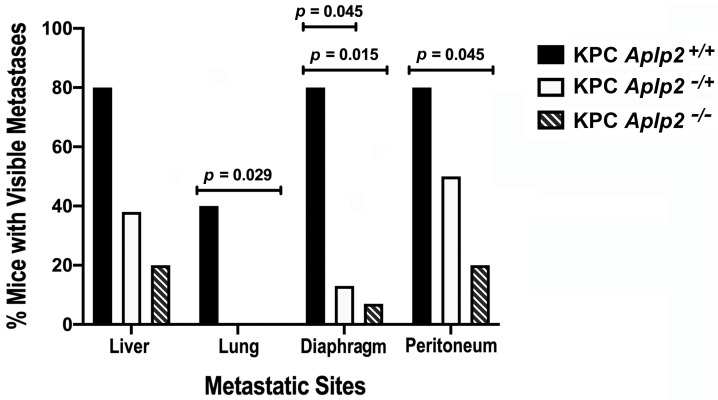
Metastatic pancreatic tumor spread was decreased upon homozygous and (to a lesser extent) heterozygous loss of APLP2 in the background of KPC. At necropsy, the liver, lung, diaphragm, and peritoneum were assessed. Mice with homozygous loss of APLP2 in the pancreas had significantly fewer metastases to the lung (*p* = 0.029), diaphragm (*p* = 0.015), and peritoneum (*p* = 0.045) than wild-type mice. Two-way ANOVA and Tukey’s multiple comparisons test were used to compare the overall metastatic spread between groups. Fisher’s Exact Test with Bonferroni Method Adjustment was used to compare specific metastatic sites between groups.

We noted a significant decrease in metastases in the KPC mice lacking APLP2 expression in the pancreas (Figure 4). At necropsy, the percentages of mice with visible lung, diaphragm, and peritoneum metastases were significantly lower among the mice with homozygous, pancreas-specific APLP2 knockout on the KPC background, relative to KPC (wild-type APLP2) mice. Furthermore, among the mice with heterozygous, pancreas-specific APLP2 knockout on the KPC background, there was a significantly lower percentage with visible metastases to the diaphragm compared to KPC (wild-type) APLP2 mice. These data indicate that APLP2 strongly influences the extent of metastasis in the KPC pancreatic cancer mouse model.

## 3. Discussion

Our work in this study has confirmed APLP2 as a highly expressed regulator of pancreatic cancer, expanding upon past research from our laboratory that indicated APLP2 has an important role in this disease [6,9,10]. Our published immunoblotting studies indicated elevated APLP2 expression in human pancreatic cancer cells, and by immunohistochemistry we had demonstrated increased APLP2 presence in primary human pancreatic tumors and metastases relative to the normal pancreas [6,10]. To obtain a comparison between the advanced and early developmental stages of pancreatic cancer, we have now also analyzed RNA-seq data from human pancreatic ductal adenocarcinoma patients and found elevated expression of APLP2 in human primary tumor epithelium when compared to either PanIN lesions or stromal cells (Figure 1, Table 1). The additional comparison of mRNA expression of pancreatic cancer tissue to healthy pancreatic tissue was not made, for the following reason. Approximately 90% of the cells in normal pancreas tissue are acinar cells, and about 90% of the mRNA transcripts in an acinar cell are from digestive pro-enzyme genes. Therefore, comparing pancreatic ductal adenocarcinoma epithelial cells to normal pancreas tissues is confounded by the extreme specialization of the vast majority of the cells in the pancreas. The use of PanINs, instead of normal pancreas tissue, as a control allows comparison to corresponding cells that are clearly committed to the pre-neoplastic lineage and yet have extremely low malignant potential. The PanIN control is more biologically meaningful than normal pancreas tissue would be as a control because it highlights the changes in expression that arise during the initial development of the tumor, rather than from dedifferentiation from a highly specialized secretory cell type. The results from our RNA-seq analysis support the concept of increased APLP2 expression throughout disease progression in human pancreatic ductal adenocarcinoma.

We had previously shown that inducible knockdown of APLP2 (via shRNA) was able to delay tumor development, reduce tumor burden, and decrease metastases to the diaphragm, kidney, and intestine in an orthotopic implantation model of pancreatic cancer [10]. In an effort to further understand APLP2′s influence on pancreatic cancer development, we have turned to autochthonous mouse models of pancreatic cancer. We performed immunohistochemistry analysis on murine KPC pancreas tissue, comparing normal tissue, early PanIN lesions, and PanIN 3/invasive carcinoma, and revealed higher expression in PanIN lesions and PanIN 3/carcinoma than in normal tissue (Figure 2).

To be able to gauge the impact of APLP2′s presence in the pancreas on the progression of pancreatic cancer, we also employed in this current study a KPC mouse model with/without expression of the *Aplp2* gene in the pancreas (i.e., conditional, pancreas-specific knockdown of APLP2). We selected the conditional KPC model to use as the basis for generating this variant as the KPC model allows for the spontaneous formation of PanIN lesions and the progression of tumors, which mimics the histopathological progression of human pancreatic cancer. Previous mouse models centered on APLP2 have shown its contribution to neurological development and processes, and knockout of APLP2 in combination with deficiencies in either APP or APLP1 (the other members of the same protein family) has been shown to be lethal [15,16]. For the generation of the KPC variant for this study, we acquired a mouse strain with conditional potential for APLP2 knockdown, and the breeding of this strain with an *FLP* recombinase produced a pseudo-wild-type mouse by removing the *lacZ/neomycin* promoter cassette. The progeny are considered “floxed” as they have a *loxP*-flanked fourth exon. Subsequent breeding of the floxed mice with a *Cre* recombinase led to the excision of exon 4, which resulted in the loss of APLP2 expression, as confirmed via immunoblotting of pancreas tissue lysates (Figure 3A).

Upon generating the conditional KPC mice with pancreas-specific APLP2 loss or deficiency (i.e., either homozygous or heterozygous knockout of APLP2), we were able to show prolonged survival in comparison to the KPC mice with wild-type APLP2 (Figure 3B). At the time that the mice were euthanized due to morbidity (according to our IACUC protocol), there were no gross differences observed at necropsy in the sizes of the primary tumors in the KPC *Aplp2^−/−^,* KPC *Aplp2^−/+^,* and KPC *Aplp2^+/+^* mice. Thus, pancreas-specific complete or partial loss of APLP2 did not entirely prevent tumor growth, although survival was prolonged by pancreas APLP2 deficiency. We observed bowel and/or biliary blockages in some of the KPC mice with all three APLP2 genotypes, which is common within the KPC model. During necropsies, we noted that all three strains of mice had large primary tumors causing pressure on the intestines and other internal organs, which in some cases resulted in rectal, urethral, or vaginal prolapses. The fact that it took longer for the KPC *Aplp2^−/+^* and KPC *Aplp2^−/−^* mice to reach the same point of morbidity (and be euthanized) as the KPC *Aplp2^+/+^* mice suggests that the primary tumors were likely growing more rapidly in the KPC *Aplp2^+/+^* mice.

A significant decrease in metastases to the lung, diaphragm, and peritoneum was observed for KPC *Aplp2^−/−^* mice compared to KPC *Aplp2^+/+^* mice (Figure 4). KPC *Aplp2^−/+^* mice also had the inhibition of metastatic spread to the diaphragm (relative to *Aplp2^+/+^* mice). Although the percentage of mice with visible metastases to the liver trended lower for KPC *Aplp2^−/−^* compared to KPC *Aplp2^−/+^* mice, and for KPC *Aplp2^−/+^* compared to KPC *Aplp2^+/+^* mice, these differences were not statistically significant. There were also no statistically significant differences in the percentages of mice with visible metastases in the lung, diaphragm, or peritoneum when KPC *Aplp2^−/−^* mice were compared with KPC *Aplp2^−/+^* mice.

Our data from human samples and mouse models point to the importance of APLP2 in cancer and signify the importance of APLP2 as a pro-tumor factor in pancreatic adenocarcinoma development. Notably, the survival of mice with either homozygous or heterozygous knockout of pancreas APLP2 on the KPC background led to similar extensions in survival. This observation indicates that even partial interference with APLP2 expression and/or function could potentially have significant therapeutic benefit in future clinical studies. These results suggest that there is a threshold level of APLP2 in the KPC mouse pancreas that facilitates tumor pathogenicity, and falling below that threshold to only half that level (i.e., to the heterozygous level) impairs APLP2′s ability to exercise its pro-tumor effects. One of the precedents for our observation that heterozygous and homozygous gene deletion leads to similar cancer phenotypes is a study in the prostate cancer field [17]. Hafeez et al. reported that either heterozygous or homozygous deletion of protein kinase C epsilon (PKCε) was capable of reducing the development and metastasis of prostate cancer in the FVB/N TRAMP mouse model [17]. Tumor weights for the heterozygous and homozygous knockout mice were significantly different from the TRAMP mice, but not from each other [17]. Therefore, partial loss of the expression of another protein (i.e., PKCε) was also able to impact phenotype in a mouse tumor model to a similar extent as full loss, as we have seen with APLP2. Our results with the novel KPC *Aplp2^−/−^,* KPC *Aplp2^−/+^,* and KPC *Aplp2^+/+^* mouse strains will promote the pursuit of additional studies to validate APLP2 as a target for utilization in new efforts to treat pancreatic cancer.

## 4. Materials and Methods

### 4.1. RNA-Seq

The process used for gene expression analysis of human pancreatic tumor epithelial cells and stromal cells has been reported [13,14]. Frozen pancreatic adenocarcinoma tissue samples that had intact RNA were selected. Prior to sample processing, target lesions were verified by a pathologist specializing in gastrointestinal cancers. The tissues were then microdissected, and cell type-specific markers were used to confirm the complete separation of epithelial and stromal samples. A total of 229 epithelial samples were analyzed: 203 from primary tumors and 26 from low-grade PanINs. The number of stromal samples assessed was 125 (each with matched epithelium), including 102 from primary tumors and 23 from low-grade PanIN samples. Paired samples of isolated epithelial cells and stromal cells were prepared for each patient (>1000 cells/sample). Libraries were generated using the Ovation RNA-Seq System V2 Kit (NuGEN, San Carlos, CA, USA), and the cDNAs were sequenced with a HiSeq 2000 (Illumina, San Diego, CA, USA) (3500 to 30,000,000 100-base-pair single-end reads). The y-axis shows log2 Transcripts Per Million (TPM), calculated by dividing the read counts for APLP2 by the length of the APLP2 genetic sequence in kb, and then dividing by a scaling factor (total reads per kb for all genes in the dataset/1,000,000). The TPM unit allows comparison of the proportion of reads mapping to a particular gene between samples. APLP2 was detected with ≥1 TPM in all samples.

### 4.2. Mice Breeding and Genotyping

The novel mouse crosses described in this report were performed according to the University of Nebraska Medical Center IACUC and U.S. Public Health Service guidelines, as well as the guidelines of the International Council for Laboratory Animal Science (ICLAS). In this project, the mice were treated in a humane manner, and in all instances, pain and discomfort to the mice were kept minimized. The mouse strain with conditional potential for APLP2 deletion (the C57BL/6N-*A^tm1BRD^ APLP2^tm2a(EUCOMM)Hmgu^*/BayMmucd strain, abbreviated as the APLP2^tm1a/tm1a^ strain) was purchased from the Mutant Mouse Resources and Research Center (MMRRC) at the University of California-Davis (Davis, CA, USA). This strain had been made using a knockout-first allele with promoter-driven cassette (KOMP) strategy (European Conditional Mouse Mutagenesis Program, EUCOMM) [18,19]. The B6.Cg-Tb(Pgk1-flpo)10Sykr/J strain (more commonly known as the FLPo-10 strain) was obtained from the University of Nebraska Medical Center Mouse Genome Engineering Core Facility (which had acquired it from Jackson Laboratories, Bar Harbor, ME, USA). The *LSL-Kras^G12D/+^*, *LSL-*Trp53^R172H/+^, and *Pdx-1-Cre* strains were gifts from Dr. David Tuveson (Cold Spring Harbor Laboratory, Cold Spring Harbor, NY, USA), and these strains were extensively backcrossed to C57BL/6 by the Rachagani and Batra research group.

**Figure 5:**
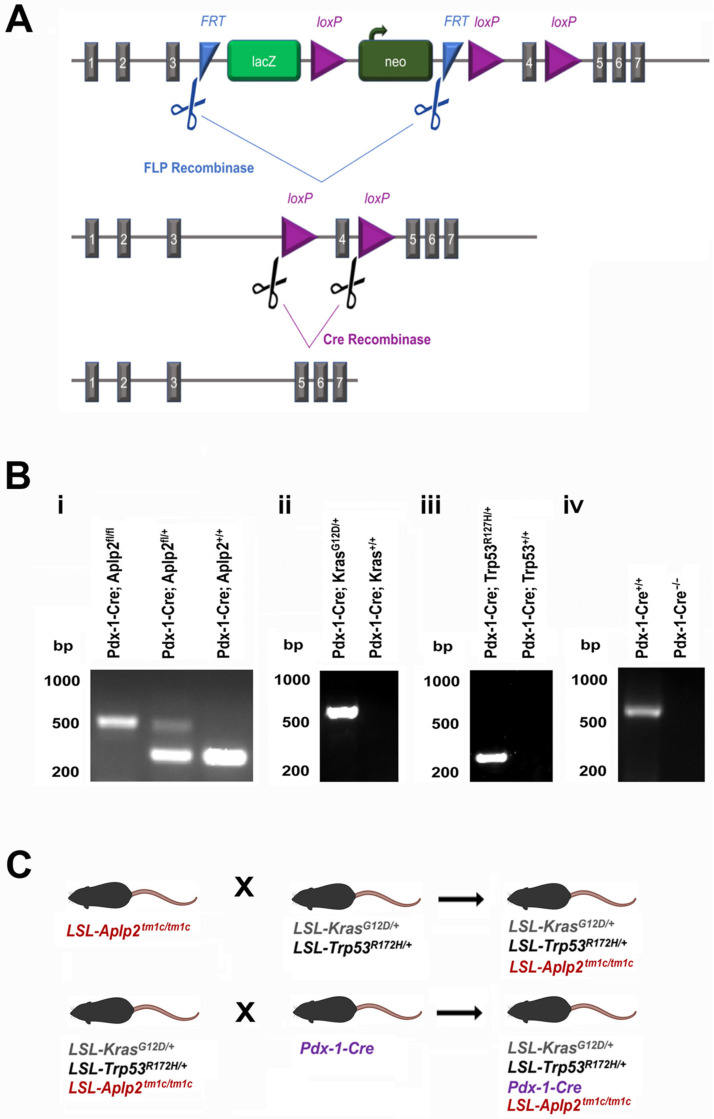
Generation of KPC variants with APLP2 deficiency in the pancreas. (**A**) The initially engineered mouse *Aplp2* construct (called *tm1a*) has FRT sites (blue triangles) flanking the *lacZ/neo* promoter cassette. Crossing mice expressing *tm1a* (*Aplp2^tm1a/tm1a^*) with Flp recombinase-expressing mice (*FLPo10*) produced mice expressing the *tm1c* construct, which lacks the *lacZ/neo* promoter cassette that had been flanked by FRT sites and which has a *loxP*-flanked fourth exon (thus, mice expressing *tm1c* are also referred to in this report as floxed *LSL-Aplp2* mice). Subsequent breeding with Cre recombinase-expressing mice (*Pdx-1-Cre*) removed the fourth exon of the *Aplp2* gene. (**B**) The mice strains were genotyped by PCR. (**i**) Co-expression of *Pdx-1-Cre* with *Aplp2^fl/fl^*, *Aplp2^fl/^*^+^, or *Aplp2^+/+^* results in differential PCR products. (**ii, iii, iv**) PCR amplification of conditional mutant (floxed) alleles resulted in a PCR product of ~600 bp for *LSL-Kras^G12D/+^* and ~250 bp for *LSL-Trp53^R172H/+^*. For *Pdx-1-Cre*, the genotyping PCR amplification resulted in a product of ~600 bp. (**C**) The stepwise mouse breeding strategy is shown.

The process of generating the KPC mice that were APLP2^+/+^, APLP2^+/−^, or APLP2^−/−^ in the pancreatic cells is shown in Figure 5. In the APLP2 EUCOMM targeting vector, the promoter-driven cassette, as well as the flippase recognition target (FRT) and loxP (excision) sites, is inserted into the APLP2 gene between exons 3 and 5 (Figure 5A). Initial crosses were set up between the *APLP2^tm2a/tm2a^* strain and the FLPo-10 mouse. This cross produced a pseudo-wild-type mouse as the *FRT*-flanked region containing the promoter-driven cassette was excised and recombined by the FLP recombinase. The *APLP2^tm2a/tm2a^* x FLPo-10 progeny are denoted as *APLP2^tm1c/+^* or floxed mice. The *APLP2^tm1c/+^* mice were backcrossed to produce the homozygous *APLP2^tm1c/tm1c^* or floxed/floxed mice. The floxed mice were then used as the founder line for the generation of knockout mice and also as a control line for experimental analysis. For consistency in nomenclature, in this report, the *APLP2^tm1c/tm1c^* or floxed/floxed mice are denoted in the same manner as the KPC strains, i.e., as *LSL-APLP2^tm1c/tm1c^* or *LSL-APLP2^tm1c/+^*.

Before the generation of quadruple mouse crosses expressing all the genetic components of the KPC mouse and pancreas-specific deficiency of APLP2, we first confirmed that crosses expressing *Pdx-1-Cre* (a construct encoding the Cre recombinase protein specifically in pancreatic cell lineages) and the floxed *tm1c* construct would have the expected loss of the *Aplp2* gene exon 4 sequence (Figure 5A). As shown in Figure 5B(i), *Pdx-1-Cre*;*Aplp2^fl/fl^*, *Pdx-1-Cre*;*Aplp2^fl/+^*, and *Pdx-1-Cre*;*Aplp2^+/+^* mice did indeed have the anticipated genetic outcomes, as revealed by detection of polymerase chain reaction (PCR) products of the appropriate sizes upon genotyping. Likewise, we verified that *Pdx-1-Cre;Kras^G12D/+^* and *Pdx-1-Cre;Kras^+/+^* mice (Figure 5B(ii)), *Pdx-1-Cre;Trp53^R172H/+^* and *Pdx-1-Cre;Trp53^+/+^* mice (Figure 5B(iii)) and *Pdx-1-Cre^+/+^* and *Pdx-1-Cre^−/−^* mice (Figure 5B(iv)) all exhibited the expected PCR genotyping results in preliminary testing.

Next, to produce quadruple mutant animals, *LSL-Aplp2 ^tmi1c/tm1c^* mice were crossed with *LSL-Kras^G12D/+^*;*LSL-Trp53*^R172H/+^ mice, followed by a crossing of *LSL-Kras^G12D/+^*;*LSL-Trp53*^R172H/+^;*LSL-Aplp2^tmi1c/tm1c^* mice with *Pdx-1-Cre* mice (Figure 5C). Thus, the *LSL-APLP2^tm1c/tm1c^* or *LSL-APLP2^tm1c/+^* mice (which have conditional potential) were interbred with the conditional *LSL-Kras^G12D/+^ LSL-Trp53^R172H/+^* mice. Finally, the *LSL-Kras^G12D/+^ LSL*-Trp53^R172H/+^ *LSL-APLP2^tm1c/+^* mice or the *LSL-Kras^G12D/+^ LSL-Trp53^R172H/+^ LSL-APLP2^tm1c/tm1c^* mice were crossed with the *Pdx-1-*Cre strain, yielding novel mutants with either homozygous or heterozygous APLP2 loss. The progeny of such crosses are identified as *LSL-Kras^G12D/+^ LSL-Trp53^R172H/+^ LSL-APLP2^tm1d/+^ Pdx-1-Cre* or as *LSL-Kras^G12D/+^ LSL-Trp53^R172H/+^ LSL- APLP2^tm1d/tm1d^ Pdx-1-Cre* animals on a C57BL/6J background.

For mouse genotyping, proteinase K in a lysis buffer consisting of 100 mM Tris (pH 8.8), 5 mM ethylenediaminetetraacetic acid (EDTA), 0.2% sodium dodecyl sulfate (SDS), and 200 mM NaCl (Fisher Scientific, Hampton, NH, USA) in H_2_O was used to extract DNA from a small portion (<5 mm) of each tail clipping that was collected from mouse pups 14–21 days of age. PCR amplification was performed using GoTaq Master Mix (Promega, Madison, WI, USA) with a set of primers for each gene of interest (*Kras*, *p53*, *Pdx-1-Cre*, and *APLP2*).

The PCR primer sequences were acquired from Eurofins Scientific (Luxembourg, Luxembourg), and their sequences are shown in Table 2. The PCR products were run on 2% agarose (VWR, Radnor, PA, USA) gels with GreenView Plus Gel Stain (GeneCopoeia, Rockville, MD, USA) in 1× Tris-acetate-ethylenediaminetetraacetic acid (EDTA) (TAE) buffer. The 1× TAE buffer is composed of 40 mM Tris, 20 mM acetic acid, and 1 mM EDTA. The gels were imaged using a Bio-Rad Gel Doc (Hercules, CA, USA).

### 4.3. Immunohistochemistry

Tissue specimens were collected from 20-week-old KPC mice (*n* = 13 total specimens, *n* = 6 normal pancreas specimens and *n* = 7 pancreatic tumors) and fixed in 10% neutral buffered formalin for 72 h at room temperature prior to embedding in paraffin (Fisher Scientific, Hampton, NH, USA). The immunohistochemical staining and analysis were performed as per a published protocol [20]. In brief, the slides were deparaffinized using xylene followed by rehydration in a series of 10-min incubations in alcohol solutions ranging from 100% to 20%. The tissues were immersed in 3% H_2_0_2_ in methanol to block endogenous peroxidase activity prior to antigen retrieval which involved incubation with 0.01 M citrate buffer (pH 6.8). The ImmPRESS Polymer Detection kit (Vector Laboratories, Inc., Burlingame, CA, USA) was used as a blocking agent for 2 h at room temperature before the sections were incubated with the primary antibody in 1% bovine serum albumin (BSA), 0.5% Tween in Tris-buffered saline (pH 7.3) at 4 °C for 24 h in a humidity chamber. The tissues were washed and then incubated with peroxidase-labeled secondary antibody (universal anti-mouse/rabbit immunoglobulin G antibody) for 30 min at room temperature. The immunostaining was completed by using a peroxidase substrate detection kit (Vector Laboratories, Inc.). The stained slides were scored, and representative pictures were taken and analyzed. Staining intensity was evaluated as follows: negative staining = 0, weak staining = 1, moderate staining = 2, and strong or intense staining = 3. A composite score was calculated as the percentage of positive cells multiplied by the intensity of staining.

### 4.4. Tissue Collection, Protein Quantification, and Immunoblotting

Mouse pancreatic tissue was collected, washed once with cold PBS, and then flash-frozen in liquid nitrogen. Cell lysis buffer was added to the tissue before processing with a mortar and pestle. The cell lysis buffer was composed of the following reagents from Sigma (St. Louis, MO, USA): 1 mM ethyleneglycol-bis(β-aminoethyl)-N,N,N′,N′-tetraacetic acid, 1 mM EDTA, 50 mM Tris-HCl pH 7.5, 1% Triton X-100, 2 mM dithiothreitol and 0.1 mM phenylmethylsulfonyl fluoride in addition to 1 mM Na_3_VO_4_ and 1 μg/mL Halt Cocktail from Thermo Fisher Scientific (Waltham, MA, USA) Following harvest, the lysates were stored at −80 °C overnight, then thawed on ice, and centrifuged in an Eppendorf 5810R centrifuge at 13,000 rpm for 30 min at 4 °C. The supernatants were transferred to new tubes and stored at −80 °C until use. All samples underwent protein quantification using the Pierce BCA Protein Assay Kit per the manufacturer’s protocol (Thermo Fisher Scientific). Aliquots of the lysate supernatants were mixed with 5× sodium dodecyl sulfate loading dye (250 mM Tris-HCl pH 6.8, 10% w/v sodium dodecyl sulfate (Tokyo Chemical Industry Company, Portland, OR, USA), 30% v/v glycerol (Sigma), 5% v/v β-mercaptoethanol (Sigma), 0.02% w/v bromophenol blue (Sigma)) and heated for 5 min at 95 °C prior to loading. The samples were loaded on 4–20% or 10–20% Invitrogen Novex Tris-glycine polyacrylamide pre-cast gels (Thermo Fisher Scientific). Electrophoresis was performed at 90 V at room temperature followed by protein transfer at 50 V for 2.5 h at room temperature to polyvinylidene difluoride Immobilon-P Millipore membranes. The membranes were blocked for 1 h in a 5% w/v solution of nonfat dry milk prior to incubation overnight at 4 °C with primary antibodies. Next, the membranes were washed 3 times with 1% Tween-20 (Thermo Fisher Scientific) in PBS for 5 min. The membranes were incubated with secondary antibodies for 1 h at room temperature before being washed 3 times for 5 min with 1% Tween-20 in PBS. The proteins were then visualized, which involved incubating the membranes in Pierce ECL Western Blotting Substrate (Thermo Fisher Scientific 32106), and imaged using the ChemiDoc MP (Bio-Rad, Hercules, CA, USA). Densitometry was performed using ImageJ.JS (ImageJ compiled into Javascript and integrated with ImJoy), freely available at https://ij.imjoy.io (accessed on 17 March 2021).

### 4.5. Statistical Analysis

Mouse survival distributions were assessed via Kaplan–Meier plots and log-rank test. Reaching a tumor volume of 1000 mm^3^ or a time period of 54 days post-treatment initiation was each designated as an experimental endpoint, and mice alive at the completion of the study were treated as censored. The log-rank test allowed for the comparison of survival distributions among groups.

Two-way ANOVA and Tukey’s multiple comparisons analysis were performed in GraphPad Prism Version 8 (GraphPad Software, San Diego, CA, USA) and were used to compare the overall metastatic spread among groups. Fisher’s Exact Test with Bonferroni Method Adjustment was used to compare individual metastatic sites among groups.

## 5. Conclusions

The comprehension of APLP2′s roles in migration and growth has been expanding, based on a mounting number of studies [6,9,10,21,22,23,24,25,26,27,28,29,30,31,32]. Our findings from this novel KPC APLP2 mouse model show a significant improvement in the survival of KPC mice with pancreas-specific, heterozygous or homozygous knockout of APLP2. The establishment of these novel mouse strains will provide numerous avenues of investigation for defining the functions of APLP2 in the development and progression of pancreatic cancer.

## Figures and Tables

**Table 1:** Differential APLP2 gene expression analysis.

Group 1	Group 2	Mean Group 1	Mean Group 2	logFC *	*t*-Statistic	FDR **
PanIN Epi ***	PanIN Stroma	9.82	9.10	−0.719	−5.37	8.87 × 10^−6^
PDA Epi ****	PDA Stroma	10.45	8.89	−1.560	−18.80	4.71 × 10^−52^
PanIn Epi	PDA Epi	9.82	10.45	0.637	3.84	1.58 × 10^−03^
PanIN Stroma	PDA Stroma	9.10	8.89	−0.211	−1.78	2.46 × 10^−1^
All Epi	All Stroma	10.39	8.93	−1.460	−19.40	6.54 × 10^−57^

* Positive log fold changes (logFC) indicate higher levels in Group 2, and negative logFC indicates lower levels; ** FDR = False Discovery Rate; *** PanIN Epithelial Cells; **** Pancreatic Ductal Adenocarcinoma Epithelial Cells.

**Table 2:** PCR primer sequences for genotyping.

Gene	Primer Sequence
*K-ras*	5′-GTCGACAAGCTCATGCGGGTG-3′
*K-ras*	5′-CCTTTACAAGCGCACGCAGACTGTAGA-3′
*K-ras*	5′-AGCTAGCCACCATGGCTTGAGTAAGTCTGCA-3′

*Trp53*	5′-CTTGGAGACATAGCCACACTG-3′
*Trp53*	5′-AGCTAGCCACCATGGCTTGAGTAAGTCTGCA-3′
*Trp53*	5′-TTACACATCCAGCCTCTGTGG-3′

*Pdx-1-Cre*	5′-CTGGACTACAATCTTGAGTTGC-3′
*Pdx-1-Cre*	5′-GGTGTACGGTCAGTAAATTTG-3′

*APLP2*	5′-ACATTTCCTGGCTACAATCCTGTGC-3′
*APLP2*	5′-ATTATTAGACTTGGCAGGCATGCTG-3′

## Data Availability

The data relevant to this study are all contained within this main article and its supplementary material.

## Supplementary Materials

The following are available online at https://www.mdpi.com/article/10.3390/cancers13071535/s1, Figure S1: Original immunoblot corresponding to Figure 3A, Table S1: APLP2 Expression Values for Each Sample, Table S2: Densitometry Readings and Intensity Ratios for Figure S1 and Figure 3A.

## Institutional Animal Care and Use Committee Statement

This project was conducted in accordance with the University of Nebraska Medical Center IACUC Protocol #17-141-12-EP (approved 27 December 2017).
