# Supplementary material for: Amyloid Precursor-like Protein 2 Expression Increases during Pancreatic Cancer Development and Shortens the Survival of a Spontaneous Mouse Model of Pancreatic Cancer
Source: Cancers (Basel). 2021 Mar 26;13(7):1535. doi: 10.3390/cancers13071535 (PMC8036577; doi:10.3390/cancers13071535)
Supplement: Supplementary file 1 [file cancers-13-01535-s001.pdf]

cancers

Supplementary Material: Amyloid Precursor-like Protein 2 Expression Increases during Pancreatic Cancer Development and Shortens the Survival of a Spontaneous Mouse Model of Pancreatic Cancer

Brittany J. Poelaert, Shelby M. Knoche, Alaina C. Larson, Poomy Pandey, Parthasarathy Seshacharyulu, Nuzhat Khan, H. Carlo Maurer, Kenneth P. Olive, Yuri Sheinin, Rizwan Ahmad, Amar B. Singh, Surinder K. Batra, Satyanarayana Rachagani and Joyce C. Solheim

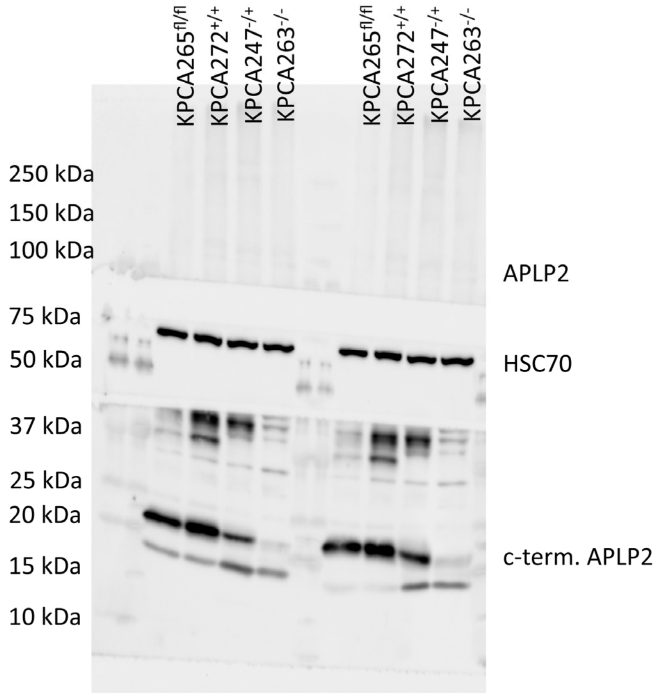

Figure S1. Original immunoblot corresponding to Figure 3A. Pancreas tissue lysates from from the KPCA265$^{fl/fl}$ (KPC *Aplp2*$^{fl/fl}$), KPCA272$^{+/+}$ (KPC *Aplp2*$^{+/+}$), KPCA247$^{-/+}$ (KPC *Aplp2*$^{-/+}$), and KPCA263$^{-/-}$ (KPC *APLP2*$^{-/-}$) mice were run in duplicate sets of lanes on an Invitrogen Novex Tris-glycine polyacrylamide pre-cast gel. The separated proteins were transferred to an Immobilon-P Millipore membrane, and each section of the membrane (top, middle, and bottom) was probed with a different antibody. Probing of the top section with the anti-APLP2 antibody was unsuccessful, but immunoblotting for HSC70 as a loading control (middle section) and for a carboxy-terminal (c-term.) segment of cleaved APLP2 (bottom section) resulted in visualization of protein bands. Positions of bands in the Bio-Rad molecular weight marker are shown at the left. Densitometry readings and intensity ratios are presented in Table S2.

Table 1. APLP2 expression values for each sample.

SampleID	Expression.log2TPM	Compartment	Type
HSH11	10.3	Epithelium	Primary
HSH12	8.84	Stroma	Primary
HSH13	10.5	Epithelium	Primary
HSH14	9.42	Stroma	Primary
HSH15	10.2	Epithelium	Primary
HSH16	8.25	Stroma	Primary
HSH17	10.7	Epithelium	Primary
HSH18	8.89	Stroma	Primary
HSH1	8.45	Epithelium	Primary
HSH2	8.95	Stroma	Primary
HSH3	9.32	Epithelium	Primary
HSH4	9.2	Stroma	Primary
HSH5	9.67	Epithelium	Primary
HSH6	9.07	Stroma	Primary
KC007	10.8	Epithelium	Primary
KC008	10	Epithelium	Primary
KC009	9.95	Epithelium	Primary
KC010	10.7	Epithelium	Primary
KC011	11.4	Epithelium	Primary
KC012	9.15	Stroma	Primary
KC013	10.7	Epithelium	Primary
KC014	7.53	Stroma	Primary
KC015	10.1	Epithelium	Primary
KC016	8.24	Stroma	Primary
KC017	9.86	Epithelium	Primary
KC018	8.3	Stroma	Primary
KC019	10.2	Epithelium	Primary
KC020	9.79	Epithelium	Primary
KC021	9.29	Stroma	Primary
KC022	9.1	Epithelium	Primary
KC023	9.08	Epithelium	Primary
KC024	8.48	Stroma	Primary
KC025	9.82	Epithelium	Primary
KC026	8.6	Stroma	Primary
KC027	10.5	Epithelium	Primary
KC028	10.1	Epithelium	Primary
KC029	8.48	Stroma	Primary
KC030	9.64	Epithelium	Primary
KC031	9.05	Stroma	Primary
KC032	10	Epithelium	Primary
KC033	8.7	Stroma	Primary
KC034	9.66	Epithelium	Primary
KC035	10.8	Epithelium	Primary
KC036	10.2	Epithelium	Primary
KC037	9.44	Stroma	Primary
KC041	9.47	Epithelium	Primary
KC043	10.5	Epithelium	Primary
KC044	11.2	Epithelium	Primary
KC045	9.23	Stroma	Primary
KC046	10.1	Epithelium	Primary
KC047	8.06	Stroma	Primary
KC048	9	Epithelium	Primary
KC049	10.8	Epithelium	Primary
KC050	11.1	Epithelium	Primary

SampleID	Expression.log2TPM	Compartment	Type
KC051	9.7	Epithelium	Primary
KC052	9.8	Epithelium	Primary
KC053	9	Stroma	Primary
KC054	8.71	Stroma	Primary
KC055	8.5	Epithelium	Primary
KC056	9.51	Epithelium	Primary
KC057	9.07	Stroma	Primary
KC058	9.03	Stroma	Primary
KC059	10.7	Epithelium	Primary
KC060	11	Epithelium	Primary
KC061	10.3	Epithelium	Primary
KC062	8.32	Stroma	Primary
KC063	10.5	Epithelium	Primary
KC064	8.82	Stroma	Primary
KC065	10.3	Epithelium	Primary
KC066	8.92	Stroma	Primary
KC067	10.5	Epithelium	Primary
KC068	10.2	Stroma	Primary
KC069	10.3	Epithelium	Primary
KC070	8.7	Stroma	Primary
KC071	10.7	Epithelium	Primary
KC072	8.95	Stroma	Primary
KC073	10.6	Epithelium	Primary
KC074	8.23	Stroma	Primary
KC075	8.86	Epithelium	Primary
KC076	7.18	Stroma	Primary
KC077	10.3	Epithelium	Primary
KC078	9.05	Stroma	Primary
KC079	10.9	Epithelium	Primary
KC080	9.16	Stroma	Primary
KC081	9.68	Epithelium	Primary
KC082	7.81	Stroma	Primary
KC083	9.94	Epithelium	Primary
KC084	8.57	Stroma	Primary
KC085	9.15	Epithelium	Primary
KC086	8.22	Stroma	Primary
KC087	11	Epithelium	Primary
KC088	9.06	Stroma	Primary
KC089	9.33	Epithelium	Primary
KC090	6.7	Stroma	Primary
KC091	8.21	Epithelium	Primary
KC092	7.92	Stroma	Primary
KC093	9.56	Epithelium	Primary
KC094	8.57	Stroma	Primary
KC095	8.99	Epithelium	Primary
KC096	8.01	Stroma	Primary
KC097	10.3	Epithelium	Primary
KC098	10.8	Epithelium	Primary
KC099	8.79	Epithelium	Primary
KC100	10.1	Epithelium	Primary
KC101	10.4	Epithelium	Primary
KC102	8.85	Epithelium	Primary
KC103	10.2	Epithelium	Primary
KC104	7.67	Epithelium	Primary
KC105	8.22	Epithelium	Primary
KC106	8.24	Stroma	Primary

SampleID	Expression.log2TPM	Compartment	Type
KC107	9.57	Epithelium	PanIN
KC108	8.47	Stroma	PanIN
KC109	10.1	Epithelium	PanIN
KC110	7.78	Stroma	PanIN
KC111	9.41	Epithelium	PanIN
KC112	8.71	Stroma	PanIN
KC113	9.44	Epithelium	PanIN
KC114	8.24	Stroma	PanIN
KC115	9.02	Epithelium	PanIN
KC116	8.79	Stroma	PanIN
KC117	9.71	Epithelium	PanIN
KC118	9.39	Epithelium	PanIN
KC119	7.76	Stroma	PanIN
KC120	8.37	Epithelium	PanIN
KC121	9.86	Epithelium	Primary
KC122	7.65	Stroma	Primary
KC123	9.92	Epithelium	Primary
KC124	8.24	Stroma	Primary
KC125	8.98	Epithelium	PanIN
KC126	7.93	Stroma	PanIN
KC127	6.78	Epithelium	Primary
KC128	8.41	Stroma	Primary
KC129	11.5	Epithelium	Primary
KC130	7.4	Epithelium	Primary
KC131	8.1	Stroma	Primary
KC132	10.2	Epithelium	Primary
KC133	7.73	Stroma	Primary
KC134	9.81	Epithelium	Primary
KC135	7.65	Stroma	Primary
KC136	9.41	Epithelium	PanIN
KC137	9.95	Epithelium	Primary
KC138	9.54	Epithelium	Primary
KC139	9.87	Epithelium	PanIN
KC140	7.51	Stroma	PanIN
KC141	9.98	Epithelium	Primary
KC142	10.9	Epithelium	Primary
KC143	9.44	Stroma	Primary
KC144	10.8	Epithelium	Primary
KC145	7.75	Stroma	Primary
KC146	10.7	Epithelium	Primary
KC147	7.54	Stroma	Primary
KC148	10.6	Epithelium	Primary
KC149	9.12	Stroma	Primary
KC150	10.6	Epithelium	Primary
KC151	10	Epithelium	Primary
KC152	8.16	Stroma	Primary
KC153	9.6	Epithelium	Primary
KC154	8.42	Stroma	Primary
KC155	9.61	Epithelium	Primary
KC156	8.4	Stroma	Primary
KC157	9.3	Epithelium	Primary
KC158	8.01	Stroma	Primary
KC159	8.62	Epithelium	Primary
KC160	9.08	Stroma	Primary
KC161	8.6	Epithelium	Primary
KC162	7.51	Stroma	Primary

SampleID	Expression.log2TPM	Compartment	Type
KC163	9.42	Epithelium	Primary
KC164	7.82	Stroma	Primary
KC165	10.4	Epithelium	Primary
KC166	7.96	Stroma	Primary
KC167	8.83	Epithelium	Primary
KC168	10.3	Epithelium	Primary
KC169	8.56	Stroma	Primary
KC170	10.8	Epithelium	Primary
KC171	8.34	Stroma	Primary
KC172	9.94	Epithelium	Primary
KC173	9.08	Epithelium	Primary
KC174	8.33	Stroma	Primary
KC175	10.5	Epithelium	Primary
KC176	7.77	Stroma	Primary
KC177	9.62	Epithelium	PanIN
KC178	8.58	Stroma	PanIN
KC179	9.61	Epithelium	Primary
KC180	8.78	Stroma	Primary
KC181	10.2	Epithelium	Primary
KC182	8.17	Stroma	Primary
KC183	11.1	Epithelium	Primary
KC184	9.74	Epithelium	Primary
KC185	9.89	Epithelium	Primary
KC186	10.6	Epithelium	Primary
KC187	10.5	Epithelium	Primary
KC188	11	Epithelium	Primary
KC189	10.6	Epithelium	Primary
KC190	8.3	Stroma	Primary
KC191	10.6	Epithelium	Primary
KC192	8.79	Stroma	Primary
KC193	9.86	Epithelium	Primary
KC194	7.18	Stroma	Primary
KC195	10.4	Epithelium	Primary
KC196	10.6	Epithelium	Primary
KC197	8.19	Stroma	Primary
KC204	9.98	Epithelium	Primary
KC205	8.29	Stroma	Primary
KC206	10.7	Epithelium	Primary
KC207	7.96	Stroma	Primary
KC208	10.2	Epithelium	Primary
KC209	10.2	Epithelium	Primary
KC210	10.5	Epithelium	Primary
KC211	8.76	Stroma	Primary
KC212	10.8	Epithelium	Primary
KC213	11.1	Epithelium	Primary
KC214	8.88	Stroma	Primary
KC215	10.1	Epithelium	Primary
KC216	7.96	Stroma	Primary
KC217	10.1	Epithelium	Primary
KC218	9.44	Epithelium	Primary
KC219	9.12	Stroma	Primary
KC220	10.6	Epithelium	Primary
KC221	8.69	Stroma	Primary
KC222	11.2	Epithelium	Primary
KC223	8.59	Stroma	Primary
KC224	10.1	Epithelium	Primary

SampleID	Expression.log2TPM	Compartment	Type
KC225	7.87	Stroma	Primary
KC227	8.84	Stroma	Primary
KC228	10	Epithelium	Primary
KC235	10.2	Epithelium	Primary
KC236	9.45	Epithelium	Primary
KC237	8.53	Stroma	Primary
KC238	11	Epithelium	Primary
KC239	8.08	Stroma	Primary
KC240	10.1	Epithelium	Primary
KC241	9.56	Stroma	Primary
KC242	11.2	Epithelium	Primary
KC243	11	Epithelium	Primary
KC244	8.41	Stroma	Primary
KC245	10.3	Epithelium	Primary
KC246	8.34	Stroma	Primary
KC247	10.7	Epithelium	Primary
KC248	11.1	Epithelium	Primary
KC249	8.37	Stroma	Primary
KC250	10.9	Epithelium	Primary
KC251	9.68	Epithelium	Primary
KC252	8.05	Stroma	Primary
KC253	9.77	Epithelium	Primary
KC254	9.69	Epithelium	Primary
KC255	8.12	Stroma	Primary
KC256	9.64	Epithelium	Primary
KC257	7.99	Stroma	Primary
KC258	10.5	Epithelium	Primary
KC259	8.07	Stroma	Primary
KC260	10.5	Epithelium	Primary
KC261	8.07	Stroma	Primary
KC265	9.64	Epithelium	Primary
KC266	9.5	Epithelium	PanIN
KC267	9.03	Stroma	PanIN
KC268	9.09	Stroma	PanIN
KC269	8.36	Epithelium	PanIN
KC270	8.41	Epithelium	PanIN
KC271	9.28	Stroma	PanIN
KC272	8.38	Epithelium	PanIN
KC273	9.04	Stroma	PanIN
KC274	8.88	Stroma	PanIN
KC275	10.2	Epithelium	PanIN
KC276	8.92	Epithelium	PanIN
KC277	8.95	Stroma	PanIN
KC278	9.24	Stroma	PanIN
KC279	8.97	Epithelium	PanIN
KC280	9.58	Epithelium	PanIN
KC281	8.54	Stroma	PanIN
KC282	9.78	Epithelium	PanIN
KC283	8.49	Stroma	PanIN
KC284	10.1	Epithelium	PanIN
KC285	9.29	Stroma	PanIN
KC286	8.91	Epithelium	PanIN
KC287	8.56	Stroma	PanIN
KC288	9.04	Epithelium	PanIN
KC289	8.61	Stroma	PanIN
KC290	8.94	Epithelium	PanIN

SampleID	Expression.log2TPM	Compartment	Type
KC291	8.49	Stroma	PanIN
KC292	8.1	Epithelium	PanIN
KC293	8.84	Stroma	PanIN
KC294	10.6	Epithelium	Primary
KC295	7.85	Stroma	Primary
KC296	10.2	Epithelium	Primary
KC297	11.1	Epithelium	Primary
KC298	10.7	Epithelium	Primary
KC299	10.7	Epithelium	Primary
KC300	10.5	Epithelium	Primary
KC301	7.91	Epithelium	Primary
KC302	10	Epithelium	Primary
KC303	9.14	Epithelium	Primary
KC304	10.1	Epithelium	Primary
KC305	8.84	Epithelium	Primary
KC306	10.7	Epithelium	Primary
KC307	9.47	Epithelium	Primary
KC308	10.4	Epithelium	Primary
KC309	11.2	Epithelium	Primary
KC310	10.6	Epithelium	Primary
KC311	8.77	Epithelium	Primary
KC312	9.46	Epithelium	Primary
KC313	9.77	Epithelium	Primary
KC314	10.7	Epithelium	Primary
KC315	10.7	Epithelium	Primary
KC316	10.3	Epithelium	Primary
KC317	10.8	Epithelium	Primary
KC318	9.41	Epithelium	Primary
KC319	11	Epithelium	Primary
KC320	9.95	Epithelium	Primary
KC321	10	Epithelium	Primary
KC322	9.51	Epithelium	Primary
KC323	9.23	Epithelium	Primary
KC324	10.1	Epithelium	Primary
KC325	10.7	Epithelium	Primary
KC326	11.4	Epithelium	Primary
KC327	10.3	Epithelium	Primary
KC328	10.5	Epithelium	Primary
KC329	9.76	Epithelium	Primary
KC330	10.3	Epithelium	Primary
KC331	9.84	Epithelium	Primary
KC332	6.63	Epithelium	Primary
KC333	10.3	Epithelium	Primary
KC334	9.8	Epithelium	Primary
KC335	10.3	Epithelium	Primary
KC336	11	Epithelium	Primary
KC337	9.27	Epithelium	Primary
KC338	10.5	Epithelium	Primary
KC339	10.8	Epithelium	Primary
KC340	10.2	Epithelium	Primary
KC341	10.6	Epithelium	Primary
KC342	10.3	Epithelium	Primary
KC343	7.48	Stroma	Primary
KC344	10.2	Epithelium	Primary
KC345	10.5	Epithelium	Primary
KC346	8.44	Stroma	Primary

SampleID	Expression.log2TPM	Compartment	Type
KC347	10.6	Epithelium	Primary
KC348	8.9	Stroma	Primary
KC349	9.62	Epithelium	Primary
KC350	10.6	Epithelium	Primary
KC351	8.35	Stroma	Primary
KC352	9.99	Epithelium	Primary
KC353	10.4	Epithelium	Primary
KC354	8.51	Stroma	Primary
KC355	11.1	Epithelium	Primary
KC356	10.1	Epithelium	Primary
KC357	7.86	Stroma	Primary
KC358	9.7	Epithelium	Primary
KC359	8.67	Stroma	Primary
KC360	7.94	Epithelium	Primary
KC361	11.4	Epithelium	Primary
KC362	8.15	Stroma	Primary
KC363	9.29	Epithelium	Primary
KC364	7.97	Stroma	Primary
KC365	10.8	Epithelium	Primary
KC366	10.1	Epithelium	Primary

Table S2. Densitometry Readings and Intensity Ratios for Figure S1 and Figure 3A.

Mouse Code Number and Mouse Genotype	Densitometry Reading for C-Term. APLP2 (ImageJ.JS)	Densitometry Reading for HSC70 Loading Control (ImageJ.JS)	Intensity Ratio (C-Term. APLP2 Divided by HSC70)
KPCA265$^{fl/fl}$ (KPC *APLP2*$^{fl/fl}$)	28121.241	22193.258	1.267
KPCA272$^{+/+}$ (KPC *APLP*$^{+/+}$)	38941.484	21832.208	1.784
KPCA247$^{-/+}$ (KPC *APLP2*$^{-/+}$)	18375.785	21442.329	0.857
KPCA263$^{-/-}$ (KPC *APLP2*$^{-/-}$)	582.263	21218.915	0.027
KPCA265$^{fl/fl}$ (KPC *APLP2*$^{fl/fl}$)	31853.212	19844.551	1.605
KPCA272$^{+/+}$ (KPC *APLP2*$^{+/+}$)	38813.706	20862.966	1.860
KPCA247$^{-/+}$ (KPC *APLP2*$^{-/+}$)	26632.848	20142.329	1.322
KPCA263$^{-/-}$ (KPC *APLP2*$^{-/-}$)	1551.991	22457.007	0.069
